# Disrupted Protein Expression and Altered Proteolytic Events in Hypophosphatemic Dentin Can Be Rescued by Dentin Matrix Protein 1

**DOI:** 10.3389/fphys.2020.00082

**Published:** 2020-02-14

**Authors:** Elizabeth Guirado, Yinghua Chen, Ryan D. Ross, Youbin Zhang, Catherine Chaussain, Anne George

**Affiliations:** ^1^Department of Oral Biology, The University of Illinois at Chicago, Chicago, IL, United States; ^2^Department of Cell & Molecular Medicine, Rush University Medical Center, Chicago, IL, United States; ^3^EA2496, Faculty of Dentistry, Université de Paris, Montrouge, France; ^4^APHP, Reference Center for Rare Disorders of the Calcium and Phosphate Metabolism, OSCAR, Bretonneau Hospital PNVS, AP-HP, Paris, France

**Keywords:** dental pulp stem cells, X-linked hypophosphatemia, hypophosphatemia, dentin matrix protein 1, matrix biology

## Abstract

Dentin, one of the four mineralized tissues of the craniofacial complex, forms sequentially from the deposition of an organic matrix to the nucleation of an inorganic phase within the matrix scaffold. Several promoters and inhibitors of mineralization support and regulate mineral nucleation. Clinical and experimental evidence suggest that dentin matrix protein 1 (DMP1) and phosphate-regulating neutral endopeptidase (PHEX) cooperate and are necessary for the formation of a cohesive dentin layer. The following study investigates the effect of PHEX loss-of-function on dentin matrix formation preceding mineralization. Using the Hyp mouse, an animal model for X-linked hypophosphatemia (XLH), we identified an irregular distribution of dentin extracellular matrix proteins. Likewise, dental pulp stem cells (DPSCs) from XLH patients exhibited altered proteolytic events with disrupted extracellular matrix deposition. Further differentiation assays demonstrated that XLH DPSCs exhibited impaired matrix mineralization. Overexpression of DMP1 in XLH DPSCs restored the irregular protein processing patterns to near-physiological levels. Our results support the hypothesis that hypophosphatemia resulting from PHEX loss-of-function affects the integrity of the organization of the dentin matrix and suggests that exogenous DMP1 can restore physiological processing of matrix proteins, in addition to its canonical role in mineralization.

## Introduction

Biological mineralization depends on the proper nucleation and growth of inorganic crystals within organic matrices. The mineralization events that occur on the self-assembled organic template are dictated by the components of the matrix itself, and are driven by the protein gradients established at the mineralization front, the result of matrix protein processing. Disruptions in the processing of these matrix components negatively affects the mineralization of these tissues, as is seen with the abnormal retention of the enamel matrix protein, amelogenin, which results in enamel hypomineralization (Priyadharsini et al., 2015). During dentinogenesis the organic matrix that is deposited by the odontoblast remains a part of the living tooth, lending to its elastic-like mechanical properties. Thus, matrix integrity is imperative for the health of dentin throughout the lifetime of the tooth.

Dentinogenesis imperfecta types I, II, and III, resulting from defective collagen type 1 or dentin sialophosphoprotein (DSPP), and autosomal recessive hypophosphatemic rickets type I (ARHR1), resulting from defective dentin matrix protein 1 (DMP1), underscore the importance of the organic dentin matrix. DMP1 and DSPP belong to the small integrin-binding ligand, N-linked glycoprotein (SIBLING) family of secreted proteins that are critical for the regulation of crystal nucleation and growth within the organic matrix. Together, collagen fibrils, glycoproteins, and non-collagenous proteins such as DMP1 and DSPP are incorporated during the self-assembly of the dentin matrix for biomineralization to occur (Goldberg et al., 2011).

X-linked hypophosphatemia (XLH) is a disease phenotypically identical to ARHR1 (MacDougall et al., 2006; Martin et al., 2011). It is considered the most common hereditary form of hypophosphatemia (1 in 20,000 births), and results from the loss-of-function of the phosphate-regulating neutral endopeptidase (PHEX) enzyme (Burnett et al., 1964; “Orphanet Report Series, 2017 – Prevalence of Rare Diseases: Bibliographic Data – June 2017 – Number 1” 2017). Over 60% of XLH patients experience periodontal defects resulting in bone loss, attachment loss, and pseudopocket formation (Foster Brian et al., 2014; Foster et al., 2014; Linglart et al., 2014; Salmon et al., 2014). Furthermore, defective fusion of calcospherites in dentin generate interglobular spaces that result in mechanical failures, pulpal exposures, and recurrent tooth abscesses (Reid et al., 1989; McWhorter and Seale, 1991; Baroncelli et al., 2006).

Although a definitive function has not been ascribed to the PHEX enzyme, it is known to bind and cleave mineral-inhibiting peptides found in SIBLING proteins (Minamizaki and Yoshiko, 2015). Although PHEX mutations cause chronic elevation of bone-derived fibroblast growth factor 23 (FGF23) leading to hypophosphatemia and hypomineralization of hard tissues, it is also believed that loss of PHEX contributes locally to the accumulation of organic matrix components leading to defective mineralization (Martin et al., 2008). In fact, studies on XLH dentin mineral reveal apatite crystals with greater levels of carbonation and lower crystallinity, two qualities that further support a matrix involvement hypothesis (Coyac et al., 2018).

In this study, primary human dental pulp stem cells (DPSCs) from XLH patients and the mouse model for XLH (Hyp) were utilized to test the hypothesis that hypophosphatemia caused by PHEX mutations result in local alterations of the organic dentin matrix. Immunohistochemical techniques provide evidence for differences in the expression and localization of matrix proteins in the Hyp mouse dentin. These differences were confirmed *in vitro* using XLH-patient DPSCs. Protein expression profiles and odontogenic potential of patient DPSCs reveal a possible functional role of the ECM governing these observed changes. Given the clinical similarities between XLH and ARHR1, the biological relationship between PHEX and DMP1 (Martin et al., 2011), and DMP1’s involvement in odontoblast differentiation and dentin matrix mineralization (George et al., 1995; Narayanan et al., 2003; Wu et al., 2011), we explore DMP1’s ability to restore physiological proteolytic events resulting in mineralization of XLH dentin.

## Materials and Methods

### Isolation of Primary Human DPSCs

Primary human DPSCs were isolated as previously published (Gronthos et al., 2000). All teeth were collected after extraction according to an orthodontic treatment plan, at the” Centre de référence des maladies rares du métabolisme du phosphore et du calcium” Service d’Odontologie, Hôpital Bretonneau, Hôpitaux Universitaires Paris Nord Val de Seine (HUPNVS), filière OSCAR, AP-HP, Paris, France. All teeth (control+XLH) were collected with informed and written consent from the patients and the parents according to ethical guidelines set by the French law (Loi Bioéthique n°2004–800) and with special ethical authorization (IRB00006477 CEERB Paris Nord) and tissue and cell banking agreements for Catherine Chaussain (n°DC-2009–927, Cellule Bioéthique DGRI/A5, Ministère de l’enseignement supérieur et de la recherche, Paris, France). Teeth were extracted and decontaminated with povidone-iodine solution, then sectioned longitudinally using dental fissure burs. Pulp tissues were gently separated from the crown and root and enzymatically digested with type I collagenase (3 mg/ml; Worthington Biochem, Freehold, NJ, United States) and dispase (4 mg/ml; Boehringer Mannheim, Germany) for 1 h at 37°C. Digests were strained through a 70-μm strainer (Falcon), cells were seeded at 1.8 × 10^4^ cells/cm^2^, and maintained in standard growth conditions [Dulbecco’s Modified Eagle Medium 1g/L D-Glucose (DMEM; Invitrogen, Grand island, NY, United States)] supplemented with 10% fetal bovine serum (FBS; Invitrogen), 1% antibiotic-antimycotic 100× (Gibco/Invitrogen, Cat. 15240062), at 37°C, 5% CO_2_. Cells were detached by trypsinization when 80–90% confluent (0.05% trypsin EDTA solution Sigma-Aldrich, St. Louis, MO, United States) and re-plated at a density of 1.8 × 10^4^cells/cm^2^. Colony-forming units (aggregates of ≥50 cells) derived from dental pulp tissue averaged 22–70 colonies/10^4^ cells plated. Cells were stored in 90% FBS-10% Dimethyl sulfoxide (DMSO, Sigma, Cat. D8418), frozen at −80°C, then transferred to liquid nitrogen for later use.

### Stable Transduction of Primary Human DPSCs

The calcium phosphate transfection method was used to transfect full-length human DMP1 cDNA into low-passage 293FT cells using a lentivirus plasmid (pLenti-DMP1-GFP-2A-Puro), together with the psPAX2 (Addgene), pMD2.G (Addgene), and pHPV17 plasmids. After 48 h, virus-containing supernatant was collected and centrifuged at 872 × *g* for 10 min, followed by 19,467 × *g* for 2 h at 4°C. The pellet was resuspended in 500 μl of cold serum-free DMEM media and transferred into an Eppendorf tube. Polybrene, 5–10 μg/ml, was added at room temperature for 15–20 min. Six-well plates containing primary DPSCs at 60% confluency were washed twice with PBS and the virus/polybrene/serum-free DMEM medium (1 ml/well) was added to the 6-well plates. Cells were incubated at room temperature for 4 h, after which the virus-containing media was removed. Wells were washed twice with PBS and 3 ml of the standard condition medium was added to each well. Antibiotic selection was achieved 24–48 h later with 1 μg/ml puromycin per well. DMP1 expression was confirmed using real-time PCR and GFP-expression.

### RNA Isolation and Gene Expression Analyses

A hybrid Trizol/RNeasy protocol was followed for the isolation of total RNA. Cell pellets were lysed in 1 ml Trizol reagent, following standard protocol. The aqueous phase was transferred to a separate Eppendorf tube and the Qiagen RNeasy Mini Kit protocol was followed thereafter (Qiagen, Cat. No. 74134). Eluted RNA was quantified spectrophotometrically, 1 μg was reverse-transcribed using the Maxima First Strand cDNA Synthesis Kit (Invitrogen, Cat. No. K1671), then stored at −80°C. For real-time PCR assays, a 1:20 dilution of cDNA in nuclease-free water was used. Two housekeeping genes (GAPDH and b-actin) were used for normalization of CT values. Fold changes in expression were determined by the ΔΔCT method, all statistics were done on ΔCT values: DMP1 (forward:5′-CAGGAGAGACAGCAAGGGTGACTCT -3′, reverse: 5′-CTCT CACTGGATTCGCTGTCTGCTTG-3′), GADPH (forward: 5′-ATCCCATCACCATCTTCCAG-3′, reverse: 5′-GAGTCCTTC CACGATACCAA-3′), b-actin (forward: 5′-AAACTGGAACGGT GAAGGTG-3′, reverse: 5′-AGAGAAGTGGGGTGGCTTTT-3′).

### Protein Isolation and Western Blot Analyses

Cell pellets were resuspended in 500 μl RIPA buffer (10X RIPA buffer with protease inhibitors). Lysates were incubated on a shaker for 1 h at 4°C, after which they were centrifuged for 30 min at 19,467 × *g* to remove cell debris. Supernatant protein concentration was assessed using the Bradford assay, 20 μg total protein was resolved using a 10% SDS-PAGE gel at 180 V for 50 min, and then transferred onto PVDF membranes at 22 V overnight. Membranes were blocked in 5% dried milk in phosphate buffered saline (PBS). Primary and HRP-conjugated secondary antibodies were resuspended at the appropriate concentrations in 5% dried milk in PBS. Western blot analysis was performed with rabbit anti-Fibronectin (1:50,000, Sigma, F3648), rabbit anti-Collagen I (1:500, Abcam, ab34710), rabbit anti-dentin matrix protein 1 and rabbit anti-dentin phosphohoryn [1:1000 (Hao et al., 2009)], rabbit anti-osteopontin (1:1000, Abcam, ab8448), and mouse anti-β-actin antibodies (1:1000, Cell Signaling Technologies, cst3700S); and developed using Pierce enhanced chemiluminescence (ECL) Plus western blotting substrate (ThermoFisher, Cat. No. 32106).

### *In vitro* Odontogenic Differentiation Assay

Dental pulp stem cells were cultured for 21 days in standard or mineralization conditions [standard conditions plus ascorbic acid (0.50 mM), β-glycerophosphate (10 mM), dexamethasone (10 nM)]. At the time of staining, media was removed from the well and the monolayer was washed twice with PBS (1X PBS, Corning, Cat. No. 21-040-CV), fixed for 10 min using 3.7% formaldehyde solution in PBS (Formaldehyde, 37% by Weight, Fisher Scientific, Cat. No BP531-500; 10X PBS (pH 7.2), Gibco Cat. No. 70013032; deionized water), and thereafter washed twice again with PBS. Alizarin Red S solution (ARS, Sigma A5533, 2% in distilled water, pH adjusted to 4.2 using 10% ammonium hydroxide, 0.22 μm filtered, and stored in the dark) was added to the monolayer, and the cells were incubated in the dark for 10 min. The ARS solution was removed, and the cells were washed with PBS until excess background staining was cleared. The final PBS wash was removed, and the cells were imaged immediately using a light microscope. Statistical analysis was performed on mean percent area stained values using a one-way ANOVA, alpha = 0.05, and the Fishers Least Significant Difference approach to control for multiple comparisons.

### *In vitro* Proliferation Assay

A cell number titration was performed in a 96-well plate. Cell numbers were serially diluted from 25,000 to 3,125 cells per 100 ml. Cells were incubated at 37°C, 5% CO_2_, for 3 h (Day 0), 24 h (Day 1), 48 h (Day 2), 4, 7, and 10 days. Fresh media (50 ml) was exchanged every fourth day. Assays were performed with a 1:1 dilution of CellTiter 96^®^ AQ_ueous_ One Solution Reagant (Promega, Cat. No. G3580) with standard cell culture media. Plates were incubated for 60, 90, and 120 min, and absorbance recorded at 490 and 630 nm. Statistical analysis of wells containing 3,125 cells/100 μl at 60 min timepoints was conducted using a two-way ANOVA, alpha = 0.01, and Sidak’s multiple comparison test using Ctrl cells as comparison controls.

### Secretome Isolation and Gelatin Zymography

Dental pulp stem cells were plated at a density of 1.8 × 10^4^ cells/cm^2^ and cultured in standard or mineralization growth conditions for 24–48 h. For secretome experiments, the media was removed and replaced with fresh serum-free media. Cells were cultured in serum-free conditions for 48 h, after which supernatants were collected and dialyzed for 4 days using a 6–8 kDa molecular weight cut off membrane (Spectra/Por^®^ Dialysis Membrane, Standard RC Tubing, Cat. No. 132665). The dialyzed supernatant was frozen at −80°C prior to lyophilization, and the lyophilized powder was resuspended in 500 μl of biological grade water. Resuspended proteins (10 mg as determined by BCA assay) were resolved using gel electrophoresis on a 10% SDS-PAGE gel for Western blotting or on a 7.5% acrylamide gel containing 1.5 mg/ml gelatin for gel zymography. Zymography gels were incubated overnight at 37°C in incubation buffer (1% Triton X-100, 50 mM Tris–HCl, 5 mM CaCl_2_, 1 μM ZnCl_2_) followed by a standard Coomassie staining protocol.

### Preliminary Assessment of WT and Hyp Molars

The preliminary assessment of molars was made possible by a collaboration with Rush University Medical Center. The mouse model for XLH (Hyp) is maintained by the Jackson laboratory on a C57BL/6J background and contains a spontaneous deletion of exons 16–22 in the PHEX gene. Two-month-old male Hyp and WT mice were euthanized according to institutional guidelines (Carpenter and Ross, 2019). Their skulls were dissected and fixed in 10% neutral-buffered formalin before paraffin-embedding and histological analysis.

### Generation of Hyp Murine Model Overexpressing DMP1 at the Odontoblast

The homozygous Dspp promoter-driven Dmp1^Tg/Tg^ (DD) transgenic male mice bred on a C57BL/6 background were crossed with heterozygous Hyp^±^ (Hyp) females to generate the experimental Dspp-Dmp1^Tg/+/^Hyp^–/0^ (DDHyp) male mice. Blood was collected at the same time of day via cardiac puncture from one-month-old mice and serum phosphate levels were determined by colorimetric assay (BioVision, Cat. No. K410) revealing hypophosphatemia (Supplementary Figure S1). Mice were euthanized according to institutional guideline. The use of animal tissues followed an approved animal use protocol approved by the UIC animal care committee (Assurance number A-3460-01).

### Micro-CT Analysis of Murine Molars

Mandibles of DDHyp and control animals were hemisected at the median mandibular fissure, fixed in 10% neutral-buffered formalin, then transferred to water before scanning. The right half of the mandible was scanned in water (Scanco Model 40; Scanco Medical AG, Bassersdorf, Switzerland) using the following conditions: 10-μm resolution, 70 kV, and 114 μA with an integration time of 300 ms for each frame. Morphological analysis of the alveolar bone was conducted as previously published (Chen et al., 2016), briefly, a buccolingual 0.240 mm thickness of alveolar bone from the furcation area of the first mandibular molar was contoured. Morphological analysis of dentin was accomplished by rotating the scan to obtain a mesiodistal view of the first mandibular molar and contouring a 0.140 mm thickness of dentin from the crest of the bone to the start of the cementum. The threshold cutoff was set at 270 for all samples for 3-dimensional segmentation and for bone volumetric calculation. Statistical analysis was performed using ordinary one-way ANOVA with Tukey’s test for correction of multiple comparisons, alpha = 0.05.

### Immunohistochemical Analysis of Murine Molars

Animal skulls were dissected and fixed for 48 h in 10% formalin solution. Tissues were washed with deionized water before decalcification in 14% ethylenediaminetetraacetic acid (EDTA), disodium salt dihydrate (pH 7.4) (Fisher Scientific, Cat. No. S311) for 5 weeks, and thereafter dehydrated and embedded in Ribbon Pro paraffin (Thermo Scientific^TM^, Cat. No. 23-031557). Sections (5 μm thick, *N* = 5) were cut using a Leica RM2255 automated rotary microtome and dried overnight at 42°C on a slide warmer. Slides (Fisherbrand^TM^ Superfrost^TM^ Plus microscope slides) were stored at room temperature before deparaffinization (65°C for 1 h) and rehydration to deionized water. Antigen retrieval was performed using sodium citrate buffer (10 mM Sodium Citrate, 0.05% Tween 20, pH 6.0) and endogenous peroxidases were quenched using 3% hydrogen peroxide in methanol. Permeabilization of tissues was performed using 0.04% Triton X-100, 1% BSA (Sigma, Cat. No. A2153). Blocking was performed using 5% BSA solution for 30 min. Primary antibodies were incubated overnight with 5% BSA solution at 4°C. Secondary antibodies for rabbit or mouse IgG were supplied in the VECTASTAIN^®^ ABC HRP kit (Vector Laboratories, Cat. No. PK-4001) or the Mouse on Mouse (M.O.M.^TM^) Elite Peroxidase Kit (Vector Laboratories, Cat. No.PK-2200), respectively. The DAB Peroxidase (HRP) Substrate Kit (Vector Laboratories, Cat. No. SK-4100) was used to visualize the selected antigens. Slides were imaged with either a Leica microscope equipped with plan apo objectives (DM2000) and a Silver Stone custom image processing unit (Leica, LAS v4.5) or a Carl Zeiss AG Axio Observer D1 inverted microscope. Quantitative image analysis was performed separately for pulp and dentin, using color deconvolution on the IHC Profiler plugin for ImageJ. Statistical analysis was performed on High Positive scores by Multiple *t*-tests with the Holm-Sidak correction for multiple comparisons, alpha = 0.05.

### Picrosirius Red Staining and CT-FIRE Analysis

**FIGURE 1:**
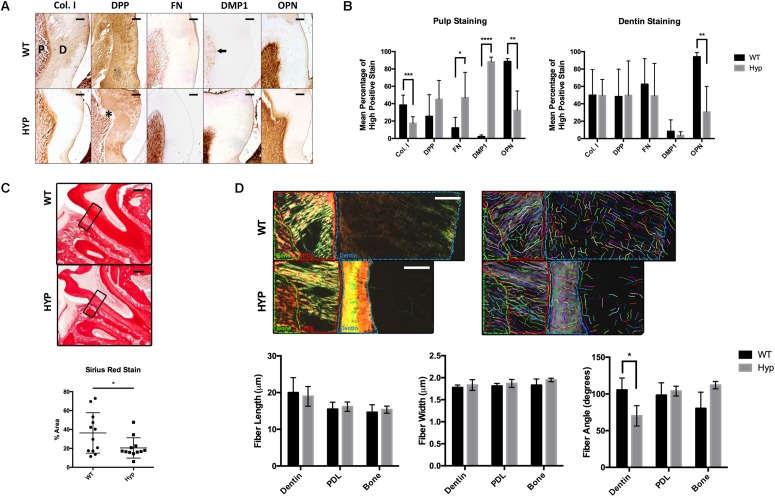
Histological comparison of WT and Hyp molars. **(A)** Immunohistochemical detection of collagen type I (Col I), dentin phosphoprotein (DPP), Fibronectin (FN), dentin matrix protein 1 (DMP1), and osteopontin (OPN) in the first mandibular molars of two-month-old wild-type (WT) and PHEX-deficient hypophosphatemic (HYP) mice. Dentin matrix (D) and pulp (P), predentin (*), mature odontoblasts (black arrow). Scale = 100 μm. **(B)** Quantitative image analysis was performed using color deconvolution on the IHC Profiler plugin for ImageJ. Statistical analysis was performed on High Positive scores by Multiple *t*-tests with the Holm-Sidak correction for multiple comparisons, alpha = 0.05. *p ≤ 0.05, **p ≤ 0.01, ***p ≤ 0.001, ****p ≤ 0.0001. **(C)** Picrosirius Red staining of collagen type I was quantified on ImageJ. Statistical analysis was performed on mean percent area stained according to a Welch’s *t*-test, alpha = 0.05. Welch’s *t*-test, *t*(16.21) = 2.292, *p* < 0.0356. **(D)** Regions of interest (dentin, PDL, and bone) within the polarized images of the Picrosirius Red stained sections were annotated and quanitfied using CT-FIRE to obtain collagen fiber length, width, and angle. Statistical analysis was performed using multiple *t*-tests with the Holm-Sidak correction for multiple comparisons, alpha = 0.05. *p ≤ 0.05, **p ≤ 0.01, ***p ≤ 0.001, ****p ≤ 0.0001. Scale = 100 μm. Boxed region of interest (ROI) on which CT-FIRE analysis was performed.

Microscope slides containing 5 μm sections were deparaffinized and rehydrated. Phosphomolybdic Acid 0.2% aqueous (Electron Microscopy Sciences, Cat. No. 26357-01, -02, -03) was added for 3 min, followed by a water rinse. Sirius Red, 0.1% in saturated Picric Acid was added to the slides for 90 min, followed by two washes in 0.01 N hydrochloric acid, dehydration, and mounting with Permount (Fisher Chemical Permount Mounting Medium, Cat. No. SP15-100). Slides were imaged using a Carl Zeiss AG Axio Observer D1 Inverted Microscope with polarizer. Bright light images were quantified, and statistical analysis was performed on mean percent area stained according to a Welch’s *t*-test, alpha = 0.05. Polarized images of the Sirius Red stained sections were analyzed using the open-source CT-FIRE software (Bredfeldt et al., 2014). Regions of interest (dentin, PDL, and bone) were annotated using the ROI manager tool and quantitative CT-FIRE analysis was performed on each to obtain collagen fiber length, width, and angle, as previously published (Chen and George, 2018). For WT versus Hyp comparisons in Figure 1, statistical analysis was performed using multiple *t*-tests with the Holm-Sidak correction for multiple comparisons, alpha = 0.05. For comparison of all genotypes (WT, DD, Hyp, and DDHyp) in Figure 4, a Two-way ANOVA with Sidak’s correction for multiple comparisons, alpha = 0.05, was utilized.

## Results

### Immunohistochemical Analysis of Wild-Type and XLH/Hyp Mouse Dentin and Pulp

The dentin and pulp of first mandibular molars from two-month-old hypophosphatemic (Hyp) mice and their C57BL/6 (WT) littermate controls were immunostained for collagen type I (Col I), dentin phosphophoryn (DPP), fibronectin (FN), dentin matrix protein 1 (DMP1), and osteopontin (OPN) (Figure 1A). Significantly weaker OPN staining was observed in Hyp dentin (M_WT_ = 94.16% high positive staining (hps), M_Hyp_ = 30.61%hps, *t*(7) = 3.60, *p* = 0.00875), although no differences in intensity were observed in dentin staining for Col I, DPP, FN, or DMP1. Despite no significant differences in Col I and DPP stain intensity between the genotypes, differences in staining pattern and localization were observed. Hyp dentin matrix was largely negative for Col I, except in the predentin region adjacent to the pulp. Patchy immunopositivity was observed in the Hyp dentin matrix for DPP, with complete absence of DPP at the predentin. WT dentin exhibited a homogenous distribution of Col I and DPP in dentin that contrasted the sparse and globular staining observed in the Hyp tooth. DPP staining was strong within the dentinal tubules of WT molars, but weak or absent from the dentinal tubules of the Hyp molars. Immunohistochemistry also revealed a wider, more defined predentin layer in Hyp dentin (^∗^). Strong immunopositivity for FN and DMP1 was observed in the pulp cells of Hyp mice when compared to WT mice. DMP1-staining in WT pulp was isolated to discrete cells adjacent to the dentin, presumably the nuclei of mature odontoblasts. At the pulp, mean staining intensity was lower for OPN (M_WT_ = 88.59% high positive staining (hps), *M*_Hyp_ = 32.41%hps, *t*(8) = 4.22, *p* = 0.00291) and Col I (*M*_WT_ = 38.43%hps, *M*_Hyp_ = 17.50%hps, *t*(15) = 4.22, *p* = 0.000741) staining were observed, along with increased FN (*M*_WT_ = 12.20%hps, *M*_Hyp_ = 46.93%hps, *t*(13) = 2.73, *p* = 0.0171) and DMP1 (*M*_WT_ = 2.15%hps, *M*_Hyp_ = 88.29%hps, *t*(6) = 26.07, *p* = 2.099e-007) staining (Figure 1B). Collagen fibers were further analyzed using Picrosirius Red staining (PRS) (Figure 1C). Hyp tooth samples stained less (*p* = 0.0356) than WT samples. Polarized light microscopy and CT-FIRE analysis revealed dentin collagen fibers that were more angled in the Hyp tooth (M_WT_ = 70.19 degrees, M_Hyp_ = 105.49 degrees, *t*(31) = 6.74, *p* = 1.540e-007). Additionally, Hyp root dentin contained bright yellow-green collagen fibers, indicative of thinner fibers, while WT dentin contained thicker orange-red fibers (Figure 1D).

### Proteolytic Activity in the Secretome of Primary Human DPSCs

**FIGURE 2:**
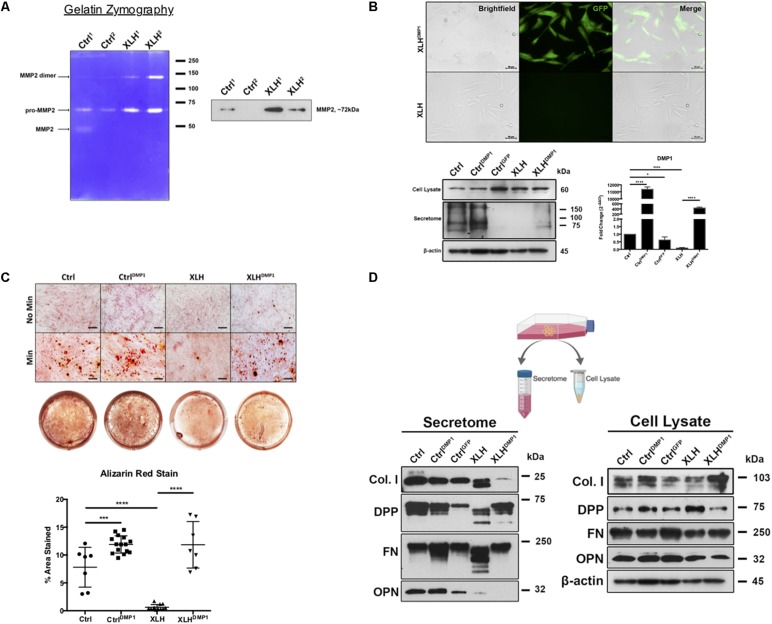
Collagenase activity in human XLH DPSC cultures. **(A)** DPSCs from two control (Ctrl1 and Ctrl2) and two XLH (XLH1 and XLH2) patients were differentiated for 2 days using mineralization conditions. Total secretome protein (5 μg) was separated on a 7.5% acrylamide gel containing gelatin. Areas of enzymatic activity appear as white bands on a blue background. Upon induction, XLH DPSCs secreted greater amounts of gelatinase MMP2 than control DPSCs. Western blot analysis revealed greater amounts of MMP2 in XLH DPSCs. MMP9 protein was undetectable by western blot (data not shown). Full-length blot available in Supplementary Material. **(B)** DPSCs were transduced with full-length human DMP1 cDNA using a lentiviral vector, pLenti-EF1α-hDMP1-GFP-2A-Puro. Representative fluorescent image GFP-positive, DMP1-overexpressing XLH DPSC cell clones (Scale = 50 μm). Ctrl DPSCs transduced with full-length human DMP1 (CtrlDMP1) and an empty vector (CtrlGFP) served as controls. Real-time PCR confirmed successful transduction and DMP1-expression in CtrlDMP1 and XLHDMP1. Fold change in gene expression was determined by the ΔΔCT method. ΔCT values were analyzed by one-way ANOVA, alpha = 0.05, *F*(4,10) = 1777, ****p < 0.0001, R2 = 0.9986, with the Sidak’s multiple comparisons test. 20 μg of total protein from cell lysates and secretome from DPSCs grown under standard conditions was analyzed by SDS-PAGE and Western blot techniques. **(C)** The odontogenic differentiation of the DPSCs was initiated using mineralization conditions (Min). Four-week cultures were stained with Alizarin Red S (ARS) to visualize calcium deposits. One-way ANOVA, alpha = 0.05. *F*(3,37) = 55.70, *p* < 0.0001, R2 = 0.8187. The Fishers Least Significant Difference approach was used to control for multiple planned comparisons. *p ≤ 0.05, ***p ≤ 0.001. Scale = 50 μm. **(D)** Western blot analysis of total protein from cell lysates (20 μg) and secretome (equal volumes) from DPSCs cultured in standard conditions (No Min). Osteopontin (OPN), dentin phosphophoryn (DPP), fibronectin (FN), and collagen type 1 (Col I). Full-length blots available in Supplementary Material, blots cropped from different gels.

Secretome derived from differentiated XLH patient samples exhibited higher levels of gelatinase activity than control samples, as evident by the intensity of MMP bands on gelatin zymography (Figure 2A). Western blot analysis of the secretome revealed the presence of MMP2 at approximately 72 kDa. These results were consistent across two independent patient samples.

### DMP1 Expression and Odontogenic Differentiation of XLH DPSCs

Ctrl and XLH DPSCs were stably transduced with full-length human DMP1 to generate Ctrl^DMP1^ and XLH^DMP1^ cell lines, respectively. Puromycin selection of DMP1-expressing, GFP-positive clones was confirmed using fluorescent microscopy (Figure 2B). Semi-quantitative gene expression of Ctrl^DMP1^ and XLH^DMP1^ cells confirmed significantly greater levels of DMP1 transcripts (*p* < 0.0001), when compared to their non-transduced counterparts. Under standard growth conditions, DMP1 expression resulted in higher levels of secreted DMP1 protein in Ctrl^DMP1^ and XLH^DMP1^ DPSCs, despite unchanged intracellular levels (Figure 2B). The effect of DMP1 expression on the odontogenic differentiation and matrix mineralization potential of XLH DPSCs was assessed by Alizarin Red S staining (Figure 2C). DMP1 expression in Ctrl and XLH DPSCs resulted in greater mineralization. Mean percent area stained values for Ctrl^DMP1^ (*M* = 11.90%, SD = 1.553) and XLH^DMP1^ (*M* = 11.84%, SD = 4.178) were significantly greater than those for Ctrl (*M* = 7.812%, SD = 3.579) and XLH (*M* = 0.6235%, SD = 0.4857), respectively. Thus, indicating an enhanced ability to initiate extracellular mineral deposition. DMP1 was able to restore the mineralization potential of XLH cells, which alone did not possess the ability to mineralize their extracellular matrix. Ctrl DPSCs stained significantly more than XLH DPSCs, and calcium deposition was absent for all cells under standard growth conditions. Proliferation assays confirmed no significant differences in proliferation rates between the cell types after 10 days in culture (Supplementary Figure S2).

### DMP1 Expression and Protein Processing in XLH DPSCs

Total protein from the cell lysates and secretome of undifferentiated cells were analyzed using Western blots. No remarkable differences were observed in the intracellular expression of Col I, FN, and OPN following DMP1-expression (Figure 2D). However, XLH^DMP1^ DPSCs possessed less intracellular DPP and more secreted DPP than XLH DPSCs, suggesting increased DPP secretion with DMP1 expression. XLH secretome-derived Col I, FN, and DPP appeared as multiple bands of lower molecular weight, suggesting additional processing of these proteins. These differences were absent from Ctrl samples. Notably, DMP1 and OPN were absent or significantly reduced in XLH secretome (Figures 2B,D). The decreased secretion of DMP1 and OPN occurred despite stable intracellular levels of these proteins. Although, DMP1 expression was sufficient to correct DPP and FN processing in XLH cells, it further decreased Col I and OPN levels.

### DMP1-Overexpression in the Murine Model of XLH

**FIGURE 3:**
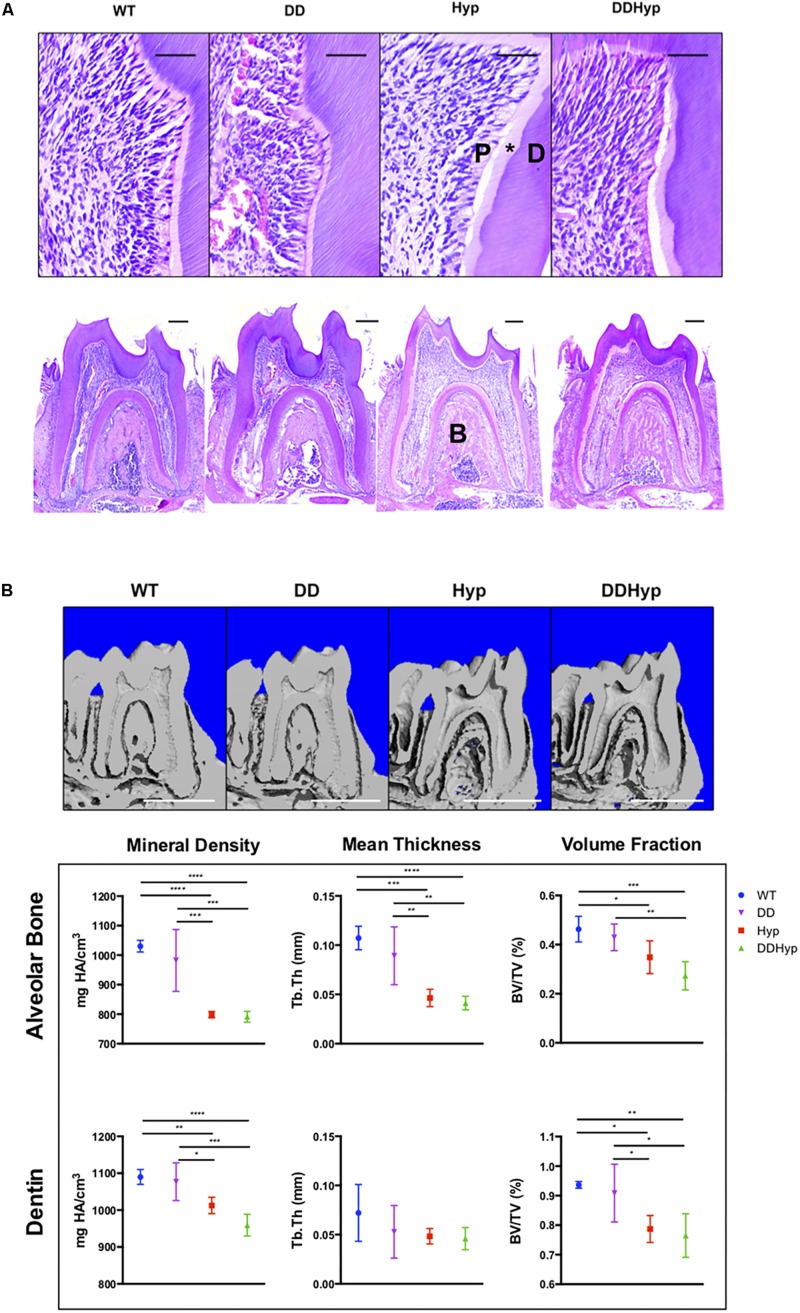
Effect of DMP1-overexpression on the murine Hyp molar. **(A)** H&E staining of one-month-old first mandibular molars from wild-type (WT), Dspp promoter-driven Dmp1Tg/Tg (DD), Hyp-/0 (Hyp), Dspp-Dmp1Tg/ + /Hyp-/0 (DDHyp) male mice. Magnified (Scale = 50 μm) and stitched panoramic image (Scale = 200 μm) containing pulp (P), predentin (*), dentin (D), and alveolar bone (B) of each genotype. Hematoxylin stain = nuclei, purple; eosin = cytoplasm, pink. **(B)** MicroCT analysis of alveolar bone and dentin from one-month-old mandibular first molars (*N* = 5). Statistical analysis conducted using an ordinary one-way ANOVA, alpha = 0.05, with Tukey’s multiple comparisons test. *p ≤ 0.05, **p ≤ 0.01, ***p ≤ 0.001, ****p ≤ 0.0001. Scale = 1 mm. Legend explains colors in graph.

Dentin sialophosphoprotein promoter-driven Dmp1^*Tg/Tg*^ (DD) transgenic male mice bred on a C57BL/6 background were crossed with heterozygous Hyp^±^ (Hyp) females to generate the experimental Dspp-Dmp1^*Tg/+/*^Hyp^–/0^ (DDHyp) male mice. Hematoxylin and eosin (H&E) staining of first mandibular molars revealed persistence of the enlarged pulp chamber and widened predentin layer in the DDHyp mouse (Figure 3A). MicroCT analysis of the alveolar bone of these animals revealed no significant differences between WT and DD or Hyp and DDHyp genotypes (Figure 3B). There were significant differences in the material density and meant trabecular thickness of WT and DD mice when compared to Hyp and DDHyp mice independently (Supplementary Table S1). No significant differences were observed between the genotypes in the thickness of root dentin, however, the material density and bone volume fraction differed significantly between Hyp and DDHyp when compared to WT and DD independently (Supplementary Table S2).

### Collagen Type I Expression in the DDHyp Model of XLH

**FIGURE 4:**
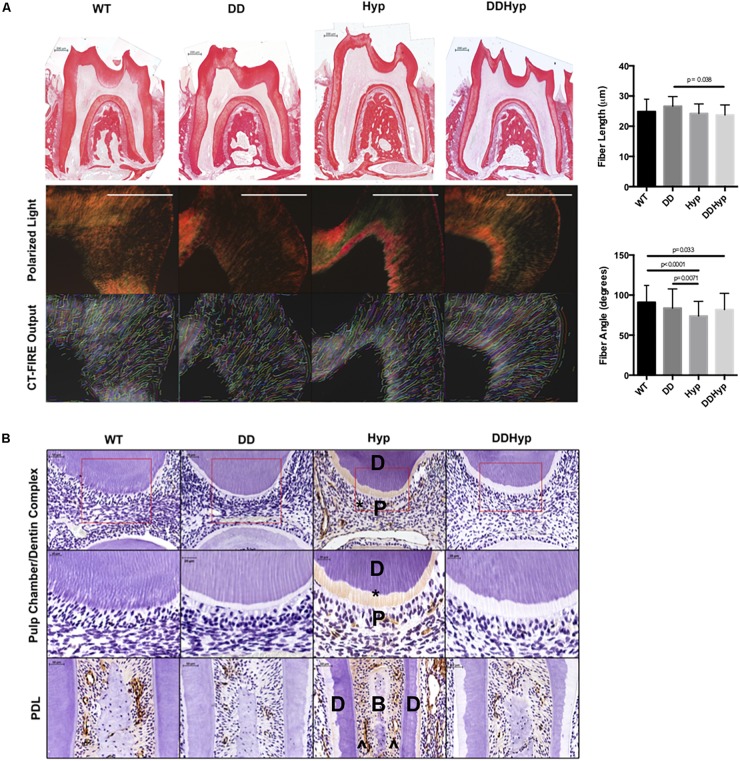
Effect of DMP1-overexpression on Collagen I, *in vivo*. **(A)** Picrosirius staining of one-month-old WT, DD, Hyp, and DDHyp mice analyzed using CT-FIRE. Statistical analysis was performed using a Two-way ANOVA, with Sidak’s correction for multiple comparisons, alpha = 0.05. Significant differences observed in fiber length and angle. No significant differences were observed in fiber width and straightness (data not shown). *N* = 5. Scale = 200 μm. **(B)** Immunohistochemical detection of collagen type I, counterstained with hematoxylin. Dentin (D); pulp (P); bone (B), predentin (*), periodontal ligament space (^). *N* = 5. Scale = 50 and 20 μm. Boxed region of ROI from which magnified subset image was taken.

Polarized light microscopy of PRS in each of the genotypes revealed significantly longer fiber lengths in the DD (*M* = 26.60 μm) versus the DDHyp (*M* = 23.73 μm) genotype. Significantly greater fiber angles were observed in WT (*M* = 90.97 degrees) when compared to Hyp (*M* = 73.81 degrees) and DDHyp (*M* = 81.80 degrees), independently. Greater fiber angles were also observed for the DD (*M* = 83.65 degrees) genotype when compared to that of the Hyp (*M* = 73.81 degrees) (Figure 4A). Immunohistochemistry revealed intense collagen type I staining in the predentin, pulp, and PDL space of the Hyp mouse (Figure 4B). Collagen staining was localized to the periodontal ligament (PDL) space of WT mice and generally reduced in the PDL space of the DD mouse. When compared to WT and Hyp genotypes, the DDHyp presented with a reduction in collagen type I staining intensity in the predentin and PDL space, similar to the DD mouse.

## Discussion

Mineralization of hard tissues depends not only on systemic factors, such as circulating phosphate and calcium levels, but on the integrity of the matrix components that are formed prior to mineral deposition and direct crystal growth. Disruption of the ECM components necessary for the template formation and mineralization of cartilage, bone, dentin, and cementum have been reported in the mouse model for XLH (*Phex*^*Hyp*^ mouse), suggesting that loss of PHEX function and/or hypophosphatemia may disrupt ECM deposition (Liang et al., 2011; Salmon et al., 2013, 2014; Coyac et al., 2017, 2018).

The current study reveals that collagen type I and several non-collagenous dentin proteins are irregularly distributed in Hyp mice, suggesting that the Hyp phenotype also consists of global alterations in the ECM. The strong FN and DMP1 staining of the Hyp pulp was surprising, since these markers are commonly seen during early stages of tooth development. In fact, the removal of FN during tooth development appears necessary for mineralization to occur (Sawada and Nanci, 1995). The decreased OPN expression in the pulp and dentin is also surprising since OPN has been reported to be elevated in the bones of Hyp mice (Zhang et al., 2010).

Dentin phosphophoryn is the most abundant non-collagenous protein in the dentin matrix with both calcium and collagen-binding ability, and has proven indispensable to the dentin mineralization process (Dahl et al., 1998). There was complete absence of DPP staining from the predentin layer of Hyp dentin and irregular, globular distribution of the protein throughout the pulp. Irregular distribution of DPP in hypophosphatemia could indicate disruption in non-collagenous protein deposition during dentinogenesis.

Immunohistochemistry revealed strong collagen type I staining in Hyp predentin, but no staining in the circumpulpal dentin, a pattern that remained consistent after using Picrosirius Red staining (PRS). PRS is a non-specific stain for a variety of collagen types that reveals information about a tissue’s collagen content, molecular order, and organization (Lattouf et al., 2014). Our group has previously used the PRS method in conjunction with polarized light microscopy to quantify the *de novo* collagen synthesis in a subcutaneous implantation model (Chen and George, 2018). In this study, polarized light microscopy revealed significant differences in the absolute angles of collagen fibers between the genotypes. The structural and/or conformational differences in the Hyp dentin collagen type I could result in the exposure of otherwise hidden MMP cleavage sites, thus contributing to the proteolytic degradation of the fibers (Chung et al., 2004). Indeed, cleavage of collagen’s C-telopeptide (25 kDa), as seen in XLH DPSC secretome samples, suggests early exposure of cleavage sites and degradation of the intact collagen triple helix (Bertassoni et al., 2012). Because all tissues were processed by the same method, the discrepancy observed between stain intensities may highlight the differences in molecular order that exist between WT and Hyp genotypes.

Although the most obvious consequence of defective PHEX enzymatic activity is the ensuing hypophosphatemic disorder, proteolytic activity appears to be central to the pathogenesis of XLH. The mechanism, however, by which the loss of one endopeptidase, PHEX, affects other proteolytic cascades is yet to be determined. Enhanced expression of cathepsin K and MMP13 have been localized to the Hyp mouse osteocyte niche (Tokarz et al., 2018). Cathepsin K is known to cleave collagens type I and II at multiple sites along the triple helix (McKleroy et al., 2013). *In vitro* studies have also reported increased cathepsin D secretion by Hyp mouse osteoblast cells (Matsumoto et al., 2005). The degradation of dentin matrix proteins by these proteases could be responsible for the aberrant proteolysis observed in this study.

The differential processing of matrix proteins during XLH odontoblast differentiation has not been reported previously. The cleavage of secreted matrix proteins, as evident by the appearance of multiple, distinct, lower molecular weight bands, occurred despite stable intracellular levels of these proteins, suggesting that secreted factors may be responsible for the observed differences. Because metalloproteases have established activity on the matrix proteins studied, we investigated their activity in our *in vitro* model (Steiglitz et al., 2004; Zhang et al., 2012; Chaussain et al., 2013). Our results demonstrated elevated levels of MMP activity following the odontogenic differentiation of hypophosphatemic dental pulp cells. It thus appears that the hypophosphatemia due to PHEX loss-of-function in odontoblasts contributes to disruptions in proteolytic activity in the tooth, as well. The effect of such disruption is unclear, since enzymatic function is necessary for the cleavage of collagenous and non-collagenous proteins during dentin development.

The irregular, unpolarized odontoblast layer found in XLH teeth indicates that a disruption in odontoblast differentiation coexists with defective dentin mineralization (Salmon et al., 2014). Cleavage products, such as the MMP2-generated C-terminal fragment of DMP1, have been implicated in the regulation of mesenchymal stem cell differentiation (Chaussain et al., 2009). Thus, it is possible that elevated levels of proteases may generate the matrix-related signaling molecules responsible for the differentiation of odontoblasts. Defective temporal regulation of stem cell differentiation could result in premature maturation, the untimely deposition of dentin matrix, and insufficiently mineralized dentin, as is the case in other models of premature odontoblast differentiation (Kim et al., 2011). Furthermore, simultaneous enhancement of proteolytic activity, as shown *in vitro* models of Hyp mouse osteoblasts (Matsumoto et al., 2005) and in our study with DPSCs, could affect the mineralization-promoting ability of these matrix proteins, resulting in the impaired mineralization that occurred in our XLH DPSC differentiation experiments.

The demonstrated response of DPP and FN in XLH^DMP1^ DPSCs, indicates that the expression of DMP1 was sufficient to correct the processing of matrix proteins secreted by XLH DPSCs. Furthermore, our attempts to translate the effects observed in the *in vitro* model following the overexpression of DMP1 to an *in vivo* model were inconclusive, perhaps due to the complexity of PHEX function during development. Despite the effects observed in collagen type I expression histologically, DMP1 expression at the odontoblast, as in the DDHyp model, did not improve the amount of tissue mineralization of either the Hyp alveolar bone or dentin. Similar approaches to this one have been attempted, albeit with global expression of the DMP1 rescue gene (Martin et al., 2012). Despite effects on circulating factors, DMP1 expression was not able to improve the Hyp phenotype. It is therefore crucial to identify the exact association between PHEX and DMP1 function during tooth development if a relationship is to be established and DMP1 is to be considered for therapeutic purposes.

In summary, our studies indicate that PHEX mutations and the resulting hypophosphatemia may negatively affect the organic matrix deposited by odontoblasts during dentinogenesis. We have shown for the first time that in XLH DPSCs the secreted extracellular matrix proteins are processed differently when compared with control cells. Altered distribution of these proteins was also seen *in vivo* in the dentin of hypophosphatemic mice. The overexpression of DMP1 was able to correct the altered processing observed in XLH cell cultures, as well as the irregular expression pattern of collagen type I in XLH mice. Together, these results implicate DMP1 in the pathogenesis of XLH. DMP1 is a developmentally regulated protein that is actively secreted by mature odontoblasts suggesting that DMP1 is an effector protein that is directly or indirectly involved in ECM protein processing and secretion (George et al., 1995). DMP1’s broad functionality merits further evaluation to determine its involvement in odontoblast differentiation, matrix deposition, and mineralization within XLH.

## Data Availability Statement

All datasets generated for this study are included in the article/Supplementary Material.

## Ethics Statement

Special Ethical authorization was received for this study (IRB00006477 CEERB Paris Nord) and tissue and cell banking agreements for Catherine Chaussain (n°DC-2009–927, Cellule Bioéthique DGRI/A5, Ministère de l’Enseignement Supérieur et de la Recherche, Paris, France). All teeth (control + XLH) were collected with informed and written consent from the patients and the parents according to ethical guidelines set by the French law (Loi Bioéthique n°2004–800).

## Author Contributions

All authors contributed to conception and design of the experiments and reviewed the manuscript. EG, YZ, CC, YC, and RR contributed to the data acquisition. AG, EG, and YC contributed to the analysis and interpretation of the results.

## Conflict of Interest

The authors declare that the research was conducted in the absence of any commercial or financial relationships that could be construed as a potential conflict of interest.
