# Supplementary material for: Disrupted Protein Expression and Altered Proteolytic Events in Hypophosphatemic Dentin Can Be Rescued by Dentin Matrix Protein 1
Source: Front Physiol. 2020 Feb 14;11:82. doi: 10.3389/fphys.2020.00082 (PMC7034300; doi:10.3389/fphys.2020.00082)
Supplement: Supplementary file 1 [file Image_1.pdf]

Supplementary Material

1 Supplementary Figures

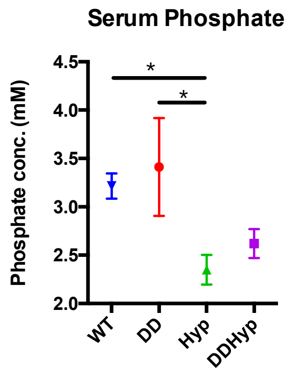

Supplementary Figure 1. Serum Phosphate Concentration. Sera from one-month-old male mice (N=5) were collected via cardiac puncture and analyzed using a colorimetric phosphate assay. WT and DD mice had significantly higher levels of serum phosphate than Hyp mice. No significant differences were observed between WT and DD, Hyp and DDHyp, WT and DDHyp, or DD and DDHyp. Statistical analysis was conducted using a two-way ANOVA, alpha = 0.05, with Sidak's multiple comparison test. * $p \leq 0.05$. Wild-type (**WT**), Dspp promoter-driven Dmp1$_{Tg/Tg}$ (**DD**), Hyp$_{-/0}$ (**Hyp**), Dspp-Dmp1$_{Tg/+}$/Hyp$_{-/0}$ (**DDHyp**).

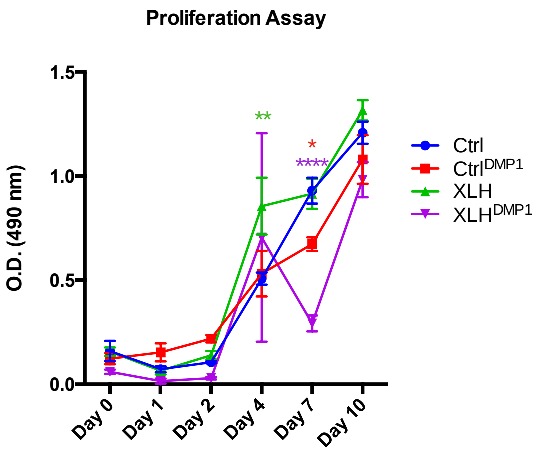

Supplementary Figure 2. *In vitro* Proliferation Assay of DPSCs. Cellular proliferation was analyzed at different timepoints during culture using a colorimetric assay. Statistical analysis was conducted using a two-way ANOVA, alpha = 0.01, with Sidak's multiple comparison test, using Ctrl cells as comparison controls. When compared to Ctrl cells, a decrease in signal was observed for XLH at Day 4, and for Ctrl$_{DMP1}$ and XLH$_{DMP1}$ at day 7. No significant differences were observed at any other time point. * $p \leq 0.05$, ** $p \leq 0.01$, **** $p \leq 0.0001$. Healthy (**Ctrl)** and **XLH** DPSCs transduced with full-length human DMP1 (**Ctrl$_{DMP1}$** and **XLH$_{DMP1}$, respectively**).
