# Supplementary material for: Disrupted Protein Expression and Altered Proteolytic Events in Hypophosphatemic Dentin Can Be Rescued by Dentin Matrix Protein 1
Source: Front Physiol. 2020 Feb 14;11:82. doi: 10.3389/fphys.2020.00082 (PMC7034300; doi:10.3389/fphys.2020.00082)
Supplement: Supplementary file 2 [file Table_1.pdf]

Table 1: Alv. Bone

Mineral Density

Tukey's multiple comparisons test	Mean Diff.	95% CI of diff.	Significant?	Summary	Adjusted P Value		
WT vs. Hyp	232.1	133.8 to 330.4	Yes	****	< 0.0001	A-B	
WT vs. DDHyp	239.1	140.8 to 337.4	Yes	****	< 0.0001	A-C	
WT vs. DD	48.23	-50.05 to 146.5	No	ns	0.5149	A-D	
Hyp vs. DDHyp	6.991	-91.29 to 105.3	No	ns	0.9969	B-C	
Hyp vs. DD	-183.9	-282.2 to -85.61	Yes	***	0.0003	B-D	
DDHyp vs. DD	-190.9	-289.2 to -92.60	Yes	***	0.0002	C-D	

Mean Thickness

Tukey's multiple comparisons test	Mean Diff.	95% CI of diff.	Significant?	Summary	Adjusted P Value	
WT vs. Hyp	0.06088	0.03044 to 0.09132	Yes	***	0.0002	A-B
WT vs. DDHyp	0.06604	0.03560 to 0.09648	Yes	****	< 0.0001	A-C
WT vs. DD	0.01808	-0.01236 to 0.04852	No	ns	0.3560	A-D
Hyp vs. DDHyp	0.00516	-0.02528 to 0.03560	No	ns	0.9613	B-C
Hyp vs. DD	-0.0428	-0.07324 to -0.01236	Yes	**	0.0049	B-D
DDHyp vs. DD	-0.04796	-0.07840 to -0.01752	Yes	**	0.0018	C-D

Volume Fraction

Tukey's multiple comparisons test	Mean Diff.	95% CI of diff.	Significant?	Summary	Adjusted P Value	
WT vs. Hyp	0.1144	0.009365 to 0.2195	Yes	*	0.0304	A-B
WT vs. DDHyp	0.1899	0.08480 to 0.2950	Yes	***	0.0005	A-C
WT vs. DD	0.03348	-0.07160 to 0.1386	No	ns	0.7990	A-D
Hyp vs. DDHyp	0.07544	-0.02964 to 0.1805	No	ns	0.2101	B-C
Hyp vs. DD	-0.08096	-0.1860 to 0.02412	No	ns	0.1642	B-D
DDHyp vs. DD	-0.1564	-0.2615 to -0.05132	Yes	**	0.0030	C-D

Table 2: Dentin

Mineral Density

Tukey's multiple comparisons test	Mean Diff.	95% CI of diff.	Significant?	Summary	Adjusted P Value	
WT vs. Hyp	77.21	17.25 to 137.2	Yes	**	0.0097	A-B
WT vs. DDHyp	130.6	70.60 to 190.5	Yes	****	< 0.0001	A-C
WT vs. DD	12.92	-47.05 to 72.89	No	ns	0.9254	A-D
Hyp vs. DDHyp	53.36	-6.609 to 113.3	No	ns	0.0902	B-C
Hyp vs. DD	-64.29	-124.3 to -4.328	Yes	*	0.0335	B-D
DDHyp vs. DD	-117.7	-177.6 to -57.69	Yes	***	0.0002	C-D

Mean Thickness

Tukey's multiple comparisons test	Mean Diff.	95% CI of diff.	Significant?	Summary	Adjusted P Value	
WT vs. Hyp	0.02374	-0.01398 to 0.06146	No	ns	0.3089	A-B
WT vs. DDHyp	0.0261	-0.01162 to 0.06382	No	ns	0.2363	A-C
WT vs. DD	0.01928	-0.01844 to 0.05700	No	ns	0.4815	A-D
Hyp vs. DDHyp	0.002360	-0.03536 to 0.04008	No	ns	0.9979	B-C
Hyp vs. DD	-0.00446	-0.04218 to 0.03326	No	ns	0.9862	B-D
DDHyp vs. DD	-0.006820	-0.04454 to 0.03090	No	ns	0.9537	C-D

Volume Fraction

Tukey's multiple comparisons test	Mean Diff.	95% CI of diff.	Significant?	Summary	Adjusted P Value	
WT vs. Hyp	0.1494	0.03052 to 0.2683	Yes	*	0.0117	A-B
WT vs. DDHyp	0.1718	0.05288 to 0.2906	Yes	**	0.0039	A-C
WT vs. DD	0.02814	-0.09074 to 0.1470	No	ns	0.9042	A-D
Hyp vs. DDHyp	0.02236	-0.09652 to 0.1412	No	ns	0.9484	B-C
Hyp vs. DD	-0.1213	-0.2401 to -0.002376	Yes	*	0.0448	B-D
DDHyp vs. DD	-0.1436	-0.2625 to -0.02474	Yes	*	0.0154	C-D
